# Trajectory of peripheral inflammation during index ECT in association with clinical outcomes in treatment-resistant depression

**DOI:** 10.1016/j.bbih.2024.100925

**Published:** 2024-12-15

**Authors:** Christina M. Hough, Jennifer L. Kruse, Randall T. Espinoza, John O. Brooks, Eliza J. Congdon, Viviane Norris, Michelle G. Craske, Katherine L. Narr

**Affiliations:** a Department of Psychology, University of California, Los Angeles (UCLA), Los Angeles, CA, USA; b Department of Psychiatry & Biobehavioral Sciences, Jane and Terry Semel Institute for Neuroscience and Human Behavior, UCLA David Geffen School of Medicine, Los Angeles, CA, USA; c Department of Neurology, Ahmanson-Lovelace Brain Mapping Center, UCLA David Geffen School of Medicine, Los Angeles, CA, USA

**Keywords:** Inflammation, Major depressive disorder, Electroconvulsive therapy, Treatment response, Treatment resistance, Affective profiles

## Abstract

**Background:**

Electroconvulsive therapy (ECT) is a highly efficacious intervention for severe and intractable depression. Evidence suggests ECT provokes an initial acute inflammatory response that subsequently decreases with repeated administration. However, relationships between inflammatory changes and clinical effects are unclear. Improved understanding of these processes may provide critical insight into effective intervention for treatment-resistant depression (TRD).

**Methods:**

Plasma inflammatory markers were assessed at pre-treatment (T1), after the second ECT session (T2), and after ECT index series completion (post-treatment/T3) in TRD (n = 40). Changes were examined over time and in association with post-treatment Responder/Non-responder status (≥50% reduction in global depression severity) and percent change in affective, cognitive and neurovegetative depressive symptom domains.

**Results:**

C-reactive protein (CRP) and interleukin-6 (IL-6) increased from pre-treatment to T2, and decreased from T2 to post-treatment. Neither early (%T2-T1) nor total (%T1-T3) change in inflammation predicted clinical outcomes, however, the interaction between early/acute inflammatory response and post-treatment inflammation (relative to baseline) was associated with clinical outcomes. Larger initial increases in IL-6 predicted greater reductions in both affective and cognitive symptoms in subjects with higher post-treatment IL-6; those with lower post-treatment IL-6 trended toward the opposite. The same was found between changes in CRP and neurovegetative symptoms.

**Conclusions:**

Though preliminary, these results demonstrate how processes involved in the acute inflammatory response to ECT may differentially influence clinical outcomes depending on overall trajectory of inflammation following ECT. Findings also highlight the importance of examining symptom-specific changes in depression when studying treatment mechanisms, rather than relying solely on global measures of severity.

## Highlights

• Index series ECT induces an initial increase in inflammation in depression.
• After the initial increase, post-treatment inflammation returns to baseline levels.
• Changes in inflammation did not predict global ECT response in depression.
• Interacting inflammatory processes relate to changes in specific depression domains.

## 1 Introduction

Despite the widespread nature and significant costs of major depression (e.g., Chisholm et al., 2016; James et al., 2018), the most commonly used treatments fail to benefit many patients. Approximately two-thirds of depressed individuals treated with first-line psychotropic or behavioral interventions are left with clinically significant symptoms (Casacalenda et al., 2002; DeRubeis et al., 2005; Driessen et al., 2013; Rush et al., 2006b; Santoft et al., 2019; Trivedi et al., 2006). Although cumulative remission rates increase with additional treatment trials, an individual's likelihood of remitting decreases with each unsuccessful trial, with a particularly large decrease following two unsuccessful treatments (Rush et al., 2006a). As such, failure to sufficiently respond to at least two adequately-dosed treatments is frequently used to define “treatment-resistant depression” (TRD) (Sackeim, 2001). In such cases, alternative interventions are often necessary. Electroconvulsive therapy (ECT) is a rapidly-acting intervention used for severe and treatment-resistant cases of major depression (Espinoza and Kellner, 2022). Compared to a variety of antidepressant medications, ECT has less drop-out and markedly improved efficacy (Kellner et al., 2016; Nygren et al., 2023; Pagnin et al., 2004; UK Ect Review Group, 2003). Despite its longtime use and impressive efficacy, however, the mechanisms of ECT's clinical effects are not well understood.

Evidence suggests that initial ECT administration acts as a potent acute stressor that activates immune and hypothalamic-pituitary-adrenal (HPA-)axis activity (Yrondi et al., 2018), including a rapid increase in C-reactive protein (CRP) and pro-inflammatory cytokines interleukin-6 (IL-6), IL-1, and tumor necrosis factor alpha (TNF-α) (Desfossés et al., 2021; Gay et al., 2021; Guloksuz et al., 2014; Lehtimäki et al., 2008; van Buel et al., 2015; Yrondi et al., 2018). This initial response appears to be short-lived, as studies involving repeated ECT have found that pro-inflammatory cytokines tend to decrease throughout treatment or over time. However, the extent of this decrease is unclear. While several studies report no differences between pre- and post-treatment (i.e., following series completion) inflammation, some have found relative decreases at post-treatment or that previously elevated basal inflammation becomes indistinguishable from healthy controls (HCs) (reviewed by Desfossés et al., 2021; Gay et al., 2021; Yrondi et al., 2018). Although many inconsistencies may be attributable to differing results among different cytokines, even findings restricted to one marker remain somewhat mixed. As such, additional research is needed to improve our understanding of acute and long-term inflammatory responses involved in ECT. Relatedly, very few studies have examined the relationship between changes in inflammation and clinical response to ECT. Some have found that long-term (i.e., pre-to post-treatment) decreases in inflammation were specific to ECT-remitters (Järventausta et al., 2017; Kranaster et al., 2018), however, others have reported no association between post-treatment changes in inflammation and changes in depression severity (Rush et al., 2016). As such, whether decreased inflammation may play a role in ECT efficacy remains unclear but worthy of further examination.

The current study aimed to assess (i) longitudinal effects of ECT on peripheral inflammation and (ii) relationships between ECT-associated changes in inflammation and clinical outcomes. To accomplish this, we first assessed levels of and changes in CRP, IL-6 and TNF-α at baseline and follow-up in HC subjects who did not undergo any intervention, compared to TRD subjects before and after ECT. As depression has been associated with increased inflammation (Dowlati et al., 2010; Enache et al., 2019; Kim et al., 2016; Leighton et al., 2018; Osimo et al., 2020) and antidepressant medications have been found to decrease pro-inflammatory cytokine levels (Więdłocha et al., 2018), we hypothesized that, compared to HC, TRD subjects would have increased basal inflammation that would decrease following ECT completion. In addition, we examined the pattern of change in inflammatory markers within TRD before beginning ECT (T1), during the early course of treatment (T2), and following index series completion (T3). We hypothesized that inflammation would increase at T2 relative to T1, followed by a decrease from T2 to T3 that would lead T3 inflammation to be decreased relative to T1.

To accomplish our second aim, we compared acute and longer-term changes in inflammation between TRD subjects classified as ECT responders versus non-responders, and also explored associations between inflammatory changes and changes in severity of depressive symptom domains. Because decreases in peripheral inflammation have previously been associated with antidepressant efficacy (Köhler et al., 2018), we hypothesized that better clinical outcomes would be associated with larger long-term decreases in inflammation. In addition, we hypothesized that the interaction between the degree of acute inflammatory change and post-treatment (T3) level of inflammation would be associated with the clinical effects of ECT. Specifically, that larger initial increases in inflammation would relate to greater decreases in depression severity (i.e., inversely correlated) when T3 inflammation is lower (i.e., more negative or less positive, relative to baseline). As depression has been associated with a blunted stress-response (Burke et al., 2005; Carroll et al., 2009, 2017; Zorn et al., 2017) and disruptions in recovery processes involved in down-regulation of inflammation and the HPA-axis (Miller et al., 2002, 2005; Pariante and Lightman, 2008), we theorized that an initial increase in inflammation may be indicative of better clinical trajectory but only in the context of intact negative feedback and reparative processes. In the absence of appropriate negative feedback processes, greater initial increases in inflammation would not be met with anti-inflammatory, anti-glucocorticoid and neuroprotective factors, and would thus lead to more chronically sustained levels of inflammation over time and worse somatic and psychological outcomes. As such, we hypothesized that clinical outcomes would be most improved for cases in which inflammation acutely increased and subsequently decreased in response to ECT. Such findings would support prior research that suggests ECT may serve to normalize both the initial stress response and its regulatory processes.

A subset (n = 29) of the present TRD sample (n = 40) was previously included in a study (Kruse et al., 2018) that examined whether changes in inflammation occur over the course of ECT (T1, T2 and T3) and whether changes in IL-6 or CRP (from T1 to T2) predicted global improvement in depression severity. They found that IL-6 and CRP (but not TNF-α or IL-8) significantly increased from T1 to T2 before returning to baseline levels by T3, and these early increases were not associated with post-treatment depression severity (Kruse et al., 2018). The present study extends and builds upon these previous findings in multiple important ways. First, due to the larger sample size, the current study is more adequately powered to detect ECT effects within the TRD group. Second, Kruse et al. (2018) did not include an HC comparison group. This addition to the current study provided the opportunity to examine cross-sectional differences in inflammatory markers between TRD and HC at T1 and T3, thus allowing us to test whether inflammation is higher in TRD at baseline and if said pro-inflammatory state “normalizes” following ECT. Further, though the present study cannot determine causality, the addition of an HC group allows us to conclude that any observed inflammatory changes in TRD after ECT exist over and above naturalistic changes occurring over time in HC. Third, this study assessed associations between changes in inflammation and changes in specific domains of depression symptoms, in addition to global symptom improvement (i.e., response status), while the only clinical outcome assessed by Kruse et al. (2018) was post-treatment global depression severity. Fourth, the current study assessed the separate *and* interactive effects of early/acute and long-term changes in inflammation on clinical outcomes, as opposed to only early/acute changes in Kruse et al. (2018). Importantly, no prior study has yet examined changes in specific depression symptom domains in relation to changes in inflammation over the course of ECT, or evaluated how the interaction between acute and long-term changes in inflammation affect clinical outcomes. As such, these novel additions have the potential to provide critical insights into the potential role of inflammatory processes in the clinical effects of ECT.

## 2 Materials and methods

### 2.1 Study subjects

Depressed subjects were recruited for participation in the current study after being referred and approved to begin ECT through Resnick Neuropsychiatric Hospital at the University of California, Los Angeles (UCLA). Healthy Controls were recruited from the greater Los Angeles community via flyers and online advertising. Data collection took place from December of 2011 through December of 2014. All participants provided written informed consent and were compensated for completion of study procedures. All study procedures were reviewed and approved by the UCLA Institutional Review Board.

Participants with TRD were determined to have met DSM-IV-TR criteria for a current major depressive episode (MDE) (80% unipolar, 20% bipolar), as determined by the Mini-International Neuropsychiatric Interview (MINI) and clinical interview with a board-certified psychiatrist. Depressed subjects had a history of two or more previous MDEs, failure to remit or respond following two or more prior adequate antidepressant medication trials during the current MDE, and initial MDE onset prior to age 50. Additional eligibility criteria included a baseline 17-item Hamilton Depression Rating Scale (HDRS) (Hamilton, 1960) rating ≥18 and baseline Montgomery-Åsberg Depression Rating Scale (MADRS) (Montgomery and Åsberg, 1977) rating ≥20. Subjects were excluded for any of the following: schizoaffective disorder, schizophrenia, alcohol/substance abuse within the prior 6-months, alcohol/substance dependence within the prior 12-months, dementia, neurological disorders, or serious medical illness. All subjects were free of psychotropic medications (including antidepressants and benzodiazepines) for at least 48–72 h prior to study entry, and had not undergone any previous trials of ECT or other neuromodulation treatment (e.g., transcranial magnetic stimulation or vagal nerve stimulation) within the prior 6-months. Additionally, HC subjects had no history of any DSM-IV-TR disorder (also confirmed by MINI) or prior psychotropic use, and were recruited to match TRD sample demographics, including age, sex, race/ethnicity and education.

A total of 72 TRD and 36 HC subjects were initially enrolled. As the primary aims of the present study related to understanding ECT mechanisms and clinical response, those without post-treatment data were not included in the present study. This left n = 46 TRD and n = 34 HC subjects. One TRD subject was excluded from analyses due to baseline depression severity being below the eligibility threshold, despite having been eligible during initial consultation. In addition, the first four TRD and eight HC subjects who were otherwise eligible did not have blood collected (at any time point), as the current study was not initially designed to include examination of inflammation markers. One additional TRD subject also did not have blood collected at any timepoint. As such, a total of n = 40 TRD and n = 26 HC subjects were included in final analyses. Of note, in addition to the study by Kruse et al. (2018) (as reviewed in the Introduction), inflammation data from TRD subjects in the present sample have been previously reported for related but distinct study aims and hypotheses (Brooks et al., 2023; Kruse et al., 2020).

### 2.2 Procedures

Patients with TRD completed morning blood draws for inflammatory measures and clinical assessments of depression symptom severity at three time points: (T1) prior to but within 24-h of their first ECT session, (T2) fewer than 24-h after completing their second ECT session but prior to beginning session three (approximately 48-h after T1), and (T3) within one week of completing their final ECT session. To mimic pre- and post-treatment (T1 and T3, respectively) data collection completed by TRD, HC subjects completed two morning blood draws approximately four-weeks apart.

### 2.3 ECT protocol

Subjects with TRD underwent ECT (5000Q MECTA Corp., Tualatin, Oregon) three times per week, using standard protocols for anesthesia (methohexital at 1 mg/kg dosage) and muscle relaxation (succinylcholine at 1 mg/kg dosage). Treatment with ECT followed the seizure threshold (ST) titration method wherein, after determining the ST (using a dose-titration method) at the first index session, ECT was administered at five-times ST for right-unilateral d’Elia lead placement using an ultra-brief pulse-width (.3 ms), and 1.5-times ST for bilateral placement using a brief pulse-width (.5 ms). Patients were routinely administered ECT using only right-unilateral lead placement, however, left-unilateral or bilateral ECT was permitted based on clinical determination (i.e., relevant ECT history or insufficient response to right-unilateral ECT) in n = 15 subjects. On average, patients completed approximately four weeks of ECT, though the exact duration of treatment varied based on clinical determination (mean = 11.5 index sessions, range = 6–22).

### 2.4 Biochemical assessments

To control for diurnal variation, blood samples were obtained between 08:00 and 11:00 a.m. Whole blood was collected in EDTA tubes and chilled before being centrifuged at 4 °C, separated into multiple aliquots, and frozen at −80 °C prior to assay. Plasma concentrations of CRP were determined by Human CRP Quantikine ELISA (R&D Systems, Minneapolis, MN). Samples were diluted 500-fold and the standard curve was extended to .4 ng/mL to obtain a lower limit of detection of .2 mg/mL, accounting for sample dilution; assay procedures otherwise followed the standard manufacturer's protocol. Mean intra-assay coefficient of variation (CV) was <3% and mean inter-assay CV was <7%. Subjects with CRP concentrations below the limit of detection (.2 mg/L) (T1: n = 5 HC and 5 TRD; T3: n = 5 HC), a value equal to one-half of the lower limit was used (.1 mg/L). When CRP concentrations were above the upper limit of the standard curve (>25 mg/L) (T2: n = 8 TRD), the estimated extrapolated CRP concentrations were utilized. Plasma concentrations of IL-6, IL-10, IL-8 and TNF-α were determined using a Bio-Plex 200 Luminex instrument and high-sensitivity immunoassay (Performance High Sensitivity Human Cytokine, R&D Systems, Minneapolis, MN), per manufacturer's protocol, including two-fold sample dilution. Mean intra-assay CV was <8% and inter-assay CV was 11–16%. All assays were performed in duplicate, with all samples from each individual subject tested on the same plate. Cytokines were measured in pg/mL; CRP was measured in mg/L.

Whereas assays for CRP were performed in a single batch, cytokines were assayed in three batches using three different test kits (batch 1: n = 15 TRD and n = 22 HC; batch 2: n = 14 TRD and n = 2 HC; batch 3: n = 11 TRD and n = 2 HC). To correct for variability between assay batches, final analyses utilized adjusted cytokine values that were calculated using regression to remove effects of assay batch.

### 2.5 Clinical assessments

Depressive symptoms were assessed in TRD subjects at T1, T2 and T3 via clinical interview using the MADRS and HDRS. The MADRS was considered the primary outcome measure of interest, however, additional exploratory analyses assessing different symptom domains of depression included individual item ratings from the HDRS. Remission status was not examined due to the limited sample size and uneven numbers of remitters (n = 10) versus non-remitters (n = 30). Instead, response status was considered the primary clinical outcome of interest. Response was defined *a priori* as ≥ 50% reduction in MADRS rating at post-treatment, relative to baseline/pre-treatment (n = 18 Responders and n = 22 Non-responders).

Given the extensive heterogeneity in depressive symptomatology, exploratory analyses were conducted to examine changes in severity of specific depressive symptom domains. Symptom domains were defined using subscales previously identified in a multi-site study of 660 depressed adults that utilized items from the MADRS, HDRS and Beck Depressive Inventory (BDI; Beck et al. (1961)) to identify three depressive symptom factors: (i) observed mood and anxiety (referred to here as “affective”), (ii) cognitive and (iii) neurovegetative (Uher et al., 2008). As the present study did not include the BDI, only items from the MADRS and HDRS were included when calculating these symptom subscales. Ratings from the individual items in each factor were summed to create three subscale scores, which were then used to index the degree of change (i.e., percent change in T3 scores relative to T1) in affective symptoms, cognitive symptoms and neurovegetative symptoms. Baseline ratings of these domains correlated with total severity ratings on the HDRS and MADRS at rho = .426 to .728 (see Supplemental Table 1 for details). Data for individual item-level ratings on the MADRS and HDRS at T1 and/or T3 were missing for n = 12 TRD patients. As such, exploratory analyses examining changes in symptom subscales were conducted on a subset of n = 28 TRD subjects.

### 2.6 Statistical analyses

To minimize multiple comparisons, analyses of inflammation were restricted to include only CRP, IL-6 and TNF-α, as these markers have been more commonly reported in prior literature regarding immunological effects of ECT (Desfossés et al., 2021; Guloksuz et al., 2014; van Buel et al., 2015; Yrondi et al., 2018) and identified in meta-analytic reviews as being increased in depression (Dowlati et al., 2010; Howren et al., 2009; Perrin et al., 2019). All analyses included age, sex.

(male/female, self-reported) and BMI as *a priori* covariates, due to their potentially confounding influence on inflammatory markers. Analyses were also conducted to determine the potential association between primary outcome variables and other demographic and clinical characteristics of diagnosis (unipolar vs. bipolar depression), race/ethnicity and years of education. As none were associated with clinical outcomes, they were not included as covariates.

Demographic and clinical data were compared between groups using independent samples t-tests, Mann-Whitney U and chi square tests, as applicable. Two-way repeated measures analysis of covariance (ANCOVA) (Group x Time) was conducted to examine the effects of diagnostic group (TRD vs. HC), time (T1 vs. T3) and their interaction, on levels of inflammatory markers. As TRD subjects completed one more study assessment than HC subjects (T2), one-way repeated measures ANCOVA was conducted to examine changes in inflammatory markers within TRD at T1, T2 and T3. Main effects and simple main effects (Bonferroni-corrected for multiple comparisons) were examined for planned comparisons to test *a priori* hypotheses. To test whether greater acute increases in inflammation predict improved clinical outcomes when accompanied by decreased inflammation at post-treatment, linear and logistic regressions were conducted within TRD. These tested the effect of the interaction between acute changes in inflammation and post-treatment inflammation levels (i.e., %T2-T1 inflammation x T3 inflammation) on clinical outcomes, including binary response status and continuous exploratory outcomes of percent change (%T3-T1) in depressive symptom subscale ratings. In addition to age, sex and BMI, these analyses also included pre-treatment inflammation level as a covariate to ensure that the pattern of change in inflammation was considered relative to pre-treatment. Prior to ANCOVA analyses, inflammatory markers were transformed using their natural logarithm to meet test assumptions of normality; the same was true for regression analyses including change in neurovegetative symptoms. All analyses were two-tailed at p < .05 and conducted using IBM SPSS (v.28.0); the PROCESS macro for SPSS (Hayes, 2017) was used to graph/visualize the results of moderation analyses.

## 3 Results

### 3.1 Sample characteristics

Demographic and clinical characteristics of HC and TRD groups are summarized in Table 1. The HC and TRD groups were well-matched in terms of demographics. There were no significant differences between these groups in terms of age, sex, BMI, race/ethnicity or years of education. Similarly, there were no differences in age, sex, BMI, race/ethnicity or years of education when comparing TRD subjects classified as ECT Responders versus Non-responders. In terms of clinical characteristics of Responders and Non-responders, there were no differences in proportion of unipolar/bipolar depression diagnosis or duration of the current MDE, however, baseline ratings of depression severity were significantly higher for ECT Responders (41.22 ± 8.63) compared to Non-responders (34. l8 ± 6.10) (t [38] = -3.017, p = .005). See Table 1 for details.

**Table 1:** Baseline demographics and clinical characteristics of the sample.

	TRD (n = 40)	HC (n = 26)	TRD vs. HC	Responders (n = 18)	Non-responders (n = 22)	Responders vs. Non-responders
Age	41.78 ± 13.73	40.50 ± 13.42	t (64) = -.372, p = .711	45.00 ± 13.09	39.14 ± 13.97	t (38) = -1.358, p = .182
Sex (female/male)	22 Female (55%) 18 Male (45%)	17 Female (65%) 9 Male (35%)	χ^2^ (1,66) = .703, p = .402	8 Female (44.4%) 10 Male (55.6%)	14 Female (63.6%) 8 Male (36.4%)	χ^2^ (1,40) = 1.473, p = .225
BMI	26.02 ± 4.76	24.62 ± 3.45	t (64) = -1.293, p = .201	27.19 ± 4.57	25.06 ± 4.81	t (38) = -1.425, p = .162
Education (years)	15.43 ± 2.72	16.65 ± 2.48	t (64) = 1.857, p = .068	14.89 ± 2.59	15.86 ± 2.80	t (38) = 1.133, p = .264
Race/Ethnicity	29 White, Non-Hispanic 5 Hispanic 2 Black 4 Asian/Pacific Islander	18 White, Non-Hispanic 4 Hispanic 2 Black 2 Asian/Pacific Islander	χ^2^ (3,66) = .401, p = .940	13 White, Non-Hispanic 3 Hispanic 1 Black 1 Asian/Pacific Islander	16 White, Non-Hispanic 2 Hispanic 1 Black 3 Asian/Pacific Islander	χ^2^ (3,40) = 1.122, p = .772
Baseline depression severity (T1 MADRS)		**--**	**--**	41.22 ± 8.63	34.l8 ± 6.10	t (38) = -3.017, p = .005∗
Current MDE Duration (years)		**--**	**--**	2.13 ± 3.17	3.03 ± 3.36 (n = 1 missing)	U = 157.5, p = .373
Diagnosis (unipolar/bipolar)		**--**	**--**	14 Unipolar (77.8%) 4 Bipolar (22.2%)	17 Unipolar (81%) 4 Bipolar (19%) (n = 1 missing)	χ^2^ (1,39) = .060, p = .807

Data are presented as Mean ± Standard Deviation unless otherwise noted.

Abbreviations: TRD = treatment-resistant depression; BMI = body mass index; MADRS = Montgomery-Äsberg Depression Rating Scale; MDE = major depressive episode.

### 3.2 Effects of ECT on inflammation

There were no significant interactive effects of Group (TRD vs. HC) by Time (T1 vs. T3) on levels of inflammation (CRP: p = .091; IL-6: p = .154; TNF-α: p = .892), however, simple main effects (Bonferroni-corrected) were examined for planned comparisons based on specific *a priori* hypotheses. Contrary to initial hypotheses, no significant differences between TRD and HC were detected in CRP, IL-6 or TNF-α levels at baseline/T1 (all p ≥ .703) or follow-up/T3 (all p ≥ .179) (see Table 2 for details). As anticipated, HC subjects did not show significant longitudinal changes in inflammation from baseline to follow-up (all p ≥ .226).

**Table 2:** Raw CRP, IL-6 and TNF-α concentrations in TRD and HC subjects.

	TRD (n = 40)	HC (n = 26)	TRD vs. HC
**Baseline (T1)**			
CRP (mg/L)	2.40 ± 3.39	1.94 ± 2.81	F (1,61) = .003, p = .958
IL-6 (pg/mL)	2.15 ± 2.11	1.56 ± .77	F (1,61) = .083, p = .775
TNF-α (pg/mL)	6.48 ± 2.56	6.30 ± 1.74	F (1,61) = .146, p = .703
**Peri-ECT (T2)**			
CRP (mg/L)	15.19 ± 20.39	–	–
IL-6 (pg/mL)	3.73 ± 6.34	–	–
TNF-α (pg/mL)	6.79 ± 2.85	–	–
**Follow-up (T3)**			
CRP (mg/L)	2.87 ± 3.83	2.81 ± 4.17	F (1,61) = 1.551, p = .218
IL-6 (pg/mL)	1.84 ± 1.58	1.88 ± .89	F (1,61) = 1.852, p = .179
TNF-α (pg/mL)	7.47 ± 6.54	6.62 ± 1.77	F (1,61) = .183, p = .671

Data are presented as Mean ± Standard Deviation unless otherwise noted.

Abbreviations: TRD = treatment-resistant depression; CRP = C-reactive protein; IL-6 = interleukin-6; TNF-α = tumor necrosis factor alpha.

**Fig. 1:**
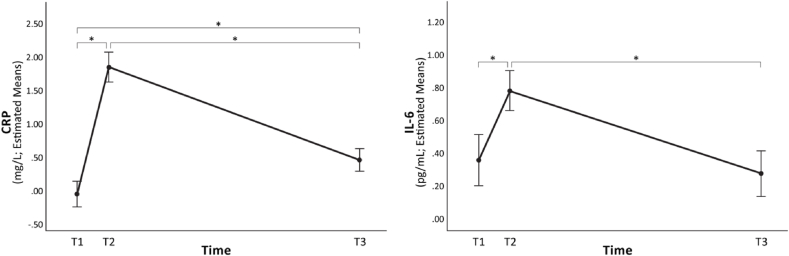
Estimated mean levels of CRP and IL-6 over time, within TRD. Levels of CRP and IL-6 significantly increased shortly after beginning ECT (T2) relative to baseline/pre-treatment (T1) (p < .001 and p = .003, respectively), followed by a significant decrease over the course of ECT (T2 to T3) (p < .001 and p = .019, respectively). Unexpectedly, post-treatment (T3) CRP levels remained significantly higher than pre-treatment/baseline (T1) (p < .001); there were no significant differences between pre- and post-treatment IL-6 levels (p > .999). Error bars represent ±1 Standard Error; estimated means calculated on average levels of covariates included in the models (age, sex and BMI).

Within TRD subjects, there was a significant main effect of Time (T1 vs. T2 vs. T3) on levels of CRP (F [1.95, 70.25] = 21.196, p < .001, ηp^2^ = .371) but not IL-6 (F [2,72] = .035, p = .965, ηp^2^ = .001) or TNF-α (F [1.783, 64.20] = .891, p = .405, ηp^2^ = .024). Planned pairwise comparisons (Bonferroni-corrected) between each time point indicated that, as hypothesized, CRP and IL-6 significantly increased shortly after beginning ECT (T2) relative to pre-treatment (T1) (p < .001 and p = .019, respectively). This increase was then followed by a significant decrease from T2 to T3 in both CRP (p < .001) and IL-6 (p = .003), however, post-treatment (T3) CRP levels remained significantly higher than baseline/pre-treatment levels (p < .001); there were no significant differences between pre- and post-treatment IL-6 levels (p > .999) (see Fig. 1). There were no changes in TNF-α concentrations over time (p ≥ .246).

### 3.3 Changes in inflammation in association with clinical outcomes

As demonstrated through separate logistic regressions, post-treatment status as an ECT Responder or Non-responder was not predicted by acute change (i.e., T2-T1 percent change) in CRP (p = .407), IL-6 (p = .443) or TNF-α (p = .389), nor was it predicted by long-term change (i.e., T3-T1 percent change) in CRP (p = .445), IL-6 (p = .304) or TNF-α (p = .258). There were also no significant relationships detected between acute or long-term changes in any inflammatory marker and changes in affective (all p ≥ .182), cognitive (all p ≥ .225) or neurovegetative (all p ≥ .138) symptoms of depression.

To test the hypothesis that the interaction between acute changes in inflammation (i.e., % T2-T1) and post-treatment levels of inflammation would predict post-treatment status as an ECT Responder/Non-responder, logistic regressions including these predictors and their interaction terms were conducted. There were no significant interactive effects detected in the models including CRP (p = .172), IL-6 (p = .231) or TNF-α (p = .277). There were, however, significant results for the models predicting changes in specific symptom subscales.

**Fig. 2:**
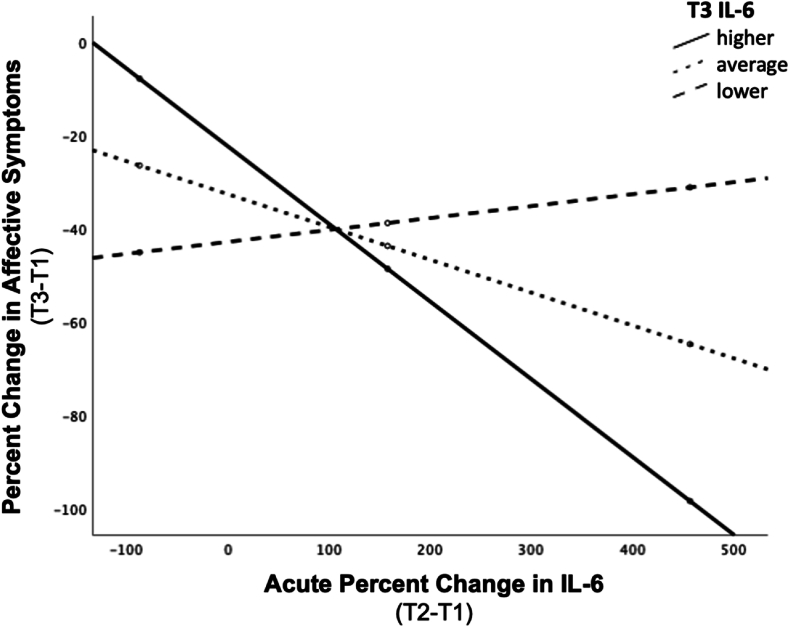
**Post-treatment (T3) IL-6 levels moderate the association between acute changes in IL-6 and changes in affective symptoms following completion of ECT.** For individuals with higher levels of IL-6 at T3, every 10% increase in IL-6 at T2 (relative to T1) was associated with a 1.7% decrease in affective symptom severity following ECT completion (relative to baseline/pre-treatment severity) (b = −.166, t [20] = -3.653, p = .002). Similarly, for subjects with average IL-6 levels at T3, every 10% increase in IL-6 at T2 relative to T1 was associated with a .7% decrease in severity of affective symptoms of depression following completion of ECT (b = −.070, t [20] = -2.835, p = .010). The association was not significant for those with lower T3 IL-6 levels (b = .026, t [20] = 1.260, p = .222).

The interaction between acute changes in IL-6 (%T2-T1) and post-treatment IL-6 levels significantly predicted changes in affective symptoms of depression (%T3-T1), over and above the effects of age, sex, BMI and baseline/pre-treatment IL-6 levels (b = −.054, t [20] = -3.833, p = .001; model summary: R^2^ = .621, F [7,20] = 4.685, p = .003). To further probe this interaction, conditional effects of acute change in IL-6 on total percent change in affective symptom severity were estimated at higher (1 standard deviation [SD] above the mean [M]), average (M), and lower (1 SD below M) levels of the moderator (post-treatment IL-6). Contrary to initial hypotheses, decreases in affective symptom severity were associated with larger acute increases in IL-6, for subjects with relatively *higher* post-treatment levels of IL-6. We can estimate that, for individuals with higher IL-6 at post-treatment, every 10% increase in concentration of IL-6 at T2 (relative to T1) was associated with a 1.7% decrease in affective symptom severity following ECT completion (relative to baseline/pre-treatment severity) (b = −.166, t [20] = -3.653, p = .002). A similar pattern was found in subjects with average post-treatment IL-6 levels, for whom, every 10% increase in IL-6 at T2 was associated with a .7% decrease in severity of affective symptoms (b = −.070, t [20] = -2.835, p = .010). The association between acute change in IL-6 and improvement in affective symptom severity was not significant for those with lower post-treatment IL-6 levels (b = .026, t [20] = 1.260, p = .222) (Fig. 2). A trend toward the same pattern was found with the interaction between acute change in CRP and post-treatment CRP levels on change in affective symptoms, though neither the overall model (R^2^ = .441, F [7,20] = 2.252, p = .073) nor the interaction term (b = −.003, t [20] = -1.932, p = .068) reached full statistical significance. The model including TNF-α did not predict change in affective symptoms (p = .162).

**Fig. 3:**
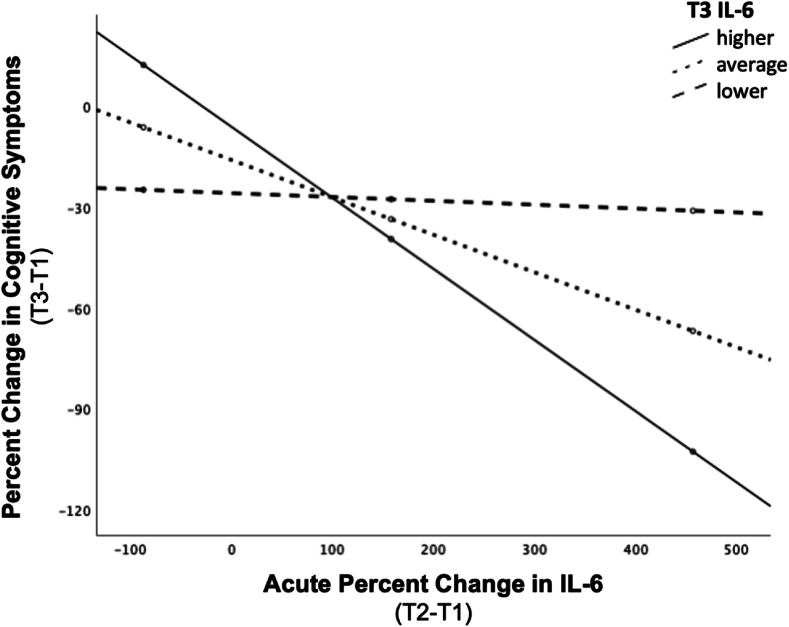
**Post-treatment (T3) IL-6 levels moderate the association between acute changes in IL-6 and changes in cognitive symptoms following completion of ECT.** For individuals with higher levels of IL-6 at T3, every 10% increase in IL-6 at T2 (relative to T1) was associated with a 2.1% decrease in cognitive symptom severity following ECT completion (relative to baseline/pre-treatment severity) (b = −.212, t [20] = -2.813, p = .011). Similarly, for subjects with average IL-6 levels at T3, every 10% increase in IL-6 at T2 relative to T1 was associated with a 1.1% decrease in severity of cognitive symptoms of depression following completion of ECT (b = −.112, t [20] = -2.720, p = .013). The association was not significant for those with lower T3 IL-6 levels (b = .012, t [20] = -.341, p = .737).

The interaction between acute change in IL-6 and post-treatment IL-6 levels significantly predicted change in cognitive symptoms of depression (% change T3-T1), over and above the effects of age, sex, BMI or baseline/pre-treatment IL-6 levels (b = −.057, t [20] = -2.419, p = .025; model summary: R^2^ = .513, F [7,20] = 3.008, p = .025). Conditional effects were probed as described above. Similar to the findings with IL-6 and change in affective symptoms, greater reductions in severity of cognitive symptoms were associated with larger acute increases in IL-6, for subjects with relatively *higher* post-treatment levels of IL-6. We can estimate that, for individuals with higher levels of IL-6 at post-treatment, every 10% increase in IL-6 at T2 was associated with a 2.1% decrease in cognitive symptom severity (b = −.212, t [20] = -2.813, p = .011). Similarly, for subjects with average levels of IL-6 at post-treatment, every 10% increase in IL-6 at T2 was associated with a 1.1% decrease in severity of cognitive symptoms of depression (b = −.112, t [20] = -2.720, p = .013). The association between acute change in IL-6 and improvement in cognitive symptom severity was not significant for those with lower post-treatment IL-6 levels (b = .012, t [20] = -.341, p = .737) (Fig. 3). The models including CRP and TNF-α did not predict changes in cognitive symptoms (p = .200 and p = .146, respectively).

**Fig. 4:**
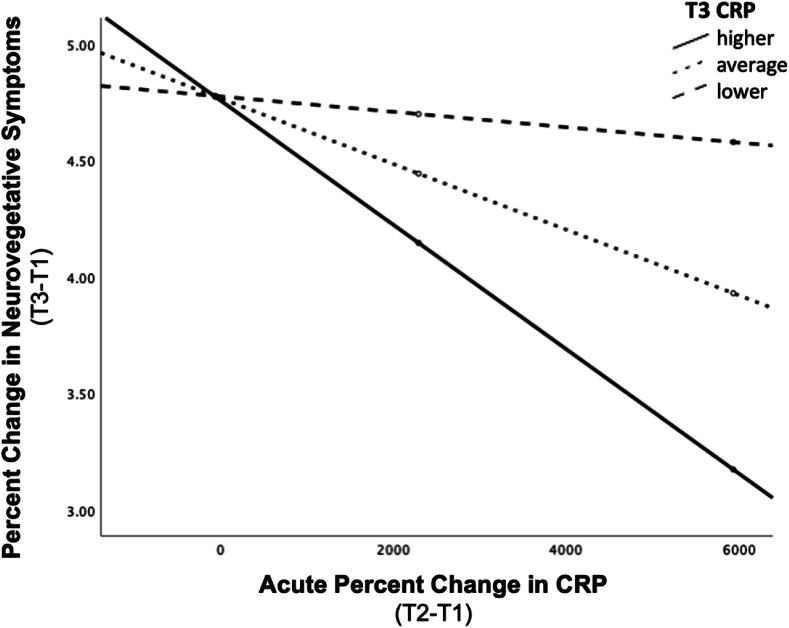
**Post-treatment (T3) CRP levels moderate the association between acute changes in CRP and changes in neurovegetative symptoms following completion of ECT.** For individuals with higher levels of CRP at T3, every 10% increase in CRP at T2 (relative to T1) was associated with a .003% decrease in neurovegetative symptom severity following ECT completion (relative to baseline/pre-treatment severity) (b = −.0003, t [20] = -3.127, p = .005). Similarly, for subjects with average CRP levels at T3, every 10% increase in CRP at T2 relative to T1 was associated with a .001% decrease in severity of neurovegetative symptoms of depression following ECT completion (b = −.0001, t [20] = -3.292, p = .004). The association was not significant for those with lower levels of T3 CRP (b = .000, t [20] = -1.023, p = .319).

Post-treatment changes in neurovegetative symptoms of depression were predicted by the pattern of change in both CRP (b = −.0001, t [20] = -2.549, p = .019; model summary: R^2^ = .695, F [7,20] = 6.513, p < .001) and TNF-α (b = −.004, t [20] = -2.167, p = .043; model summary: R^2^ = .626, F [7,20] = 4.779, p = .003), such that acute change in CRP or TNF-α differentially predicted change in neurovegetative symptoms based on post-treatment levels of CRP or TNF-α, over and above the effects of age, sex, BMI or baseline/pre-treatment CRP or TNF-α levels. For the model including CRP, conditional effects were probed similarly to that described above (i.e., at T3 CRP M ± 1 SD), however, because the lowest observed CRP level at T3 was slightly higher than 1 SD below M, the minimum observed value was used for the lower level of the moderator. Similar to the findings above, greater reductions in severity of neurovegetative symptoms were associated with larger acute increases in CRP, for subjects with relatively *higher* post-treatment CRP levels. We can estimate that, for individuals with higher levels of CRP at post-treatment, every 10% increase in CRP at T2 was associated with a .003% decrease in neurovegetative symptom severity (b = −.0003, t [20] = -3.127, p = .005). For subjects with average post-treatment CRP levels, every 10% increase in CRP at T2 was associated with a .001% decrease in severity of neurovegetative symptoms of depression (b = −.0001, t [20] = -3.292, p = .004). The association between acute change in CRP and improvement in neurovegetative symptom severity was not significant for those with lower post-treatment CRP levels (b = .000, t [20] = -1.023, p = .319). See Fig. 4. For the model including TNF-α, conditional effects were probed as previously described (i.e., at T3 TNF-α M ± 1 SD). Somewhat distinct from the relationships detected in models including IL-6 and CRP, subjects with relatively *lower* post-treatment levels of TNF-α tended to show an association between larger acute increases in T2 TNF-α and *worsening* of neurovegetative symptom severity (b = .013, t [20] = 1.929, p = .068), however, this conditional effect failed to meet thresholds for statistical significance, as did the conditional effects at average (b = .004, t [20] = .813, p = .426) and higher (b = −.004, t [20] = -.698, p = .493) post-treatment TNF-α levels. A trend similar to the relationship observed in CRP was detected for the model including IL-6 (R^2^ = .595, F [7,20] = 4.205, p = .005), however, the interaction term did not reach full statistical significance (b = −.001, t [20] = -1.746, p = .096) and so conditional effects were not subsequently examined.

## 4 Discussion

We assessed the longitudinal effects of ECT on levels of peripheral inflammation (CRP, IL-6 and TNF-α) in subjects with TRD, in comparison to HC subjects, and in association with post-treatment clinical outcomes. To the best of our knowledge, this is the first study to examine the relationship between ECT-associated changes in inflammation and changes in specific depressive symptom domains, as opposed to global clinical outcomes of depression (i.e., overall reduction in symptoms, clinical response or remission). Further, although previous studies have examined both acute and long-term changes in inflammation in association with treatment outcomes to ECT, to the best of our knowledge, none have examined how the interaction between these processes relates to clinical outcomes.

No baseline differences in inflammatory markers were detected when comparing TRD and HC groups. It is worth noting that the original study was not initially powered to detect such cross-sectional between-group differences. As such, this null result could very likely be due to insufficient power to detect a true effect. Alternatively, some have theorized that increased inflammation may not be related to all cases of depression but might instead reflect a specific (as yet undefined) depressive subtype (Raison and Miller, 2011). If that is the case, it is possible that this subtype is not well-represented in the present sample.

In alignment with initial hypotheses and prior literature, our results indicate that ECT is associated with an acute spike in inflammation shortly after beginning ECT, that then resolves over the course of treatment. Although T3 CRP remained somewhat increased compared to pre-treatment (despite decreasing after T2), it seems unlikely that this elevation reflects a persistently increased inflammatory state post-ECT series completion. Because CRP is synthesized through stimulation of hepatocytes by IL-6, changes in IL-6 tend to precede those in CRP (Heinrich et al., 1990). Given that IL-6 concentrations returned to baseline following the spike at T2, and CRP levels substantially decreased following T2, it is likely that post-treatment CRP levels follow a similar – albeit somewhat delayed – trajectory to IL-6 and would return to baseline with additional time. Though ECT series completion has previously been associated with decreases in IL-6 and other pro-inflammatory markers relative to baseline, findings are mixed and somewhat difficult to interpret due to frequent use of small sample sizes, methodological differences and varying results amongst different cytokines (reviewed in Desfossés et al., 2021; Gay et al., 2021; Yrondi et al., 2018). Previous studies have found that ECT reduced IL-6 levels (i) across all depressed subjects (Belge et al., 2020), (ii) in subjects who remitted but not in those who failed to reach remission (Järventausta et al., 2017; Kranaster et al., 2018), and (iii) when treatment included both ECT and antidepressant medication (Freire et al., 2017). On the other hand, numerous reports of no significant differences between IL-6 levels before and after ECT series completion have also been made (Fluitman et al., 2011; Kargar et al., 2014; Rotter et al., 2013; Rush et al., 2016; Ryan and McLoughlin, 2022; Schwieler et al., 2016; Zincir et al., 2016). Of those that examined TNF-α or CRP, nearly all reported no change in either of these pro-inflammatory markers following series completion (Belge et al., 2020; Fluitman et al., 2011; Kargar et al., 2014; Rotter et al., 2013; Rush et al., 2016; Ryan and McLoughlin, 2022; Schwieler et al., 2016; Sorri et al., 2018; Zincir et al., 2016), although Hestad et al. (2003) reported decreased post-treatment TNF-α levels and Freire et al. (2017) reported increased post-treatment TNF-α levels.

Though we originally predicted that larger initial increases in inflammation would predict better clinical outcomes following ECT, no such associations were detected. Given the mixed findings reported in the literature on this topic (as reviewed by Desfossés et al., 2021; Gay et al., 2021; Yrondi et al., 2018), this null result is not entirely unexpected. We similarly did not find any associations between long-term (i.e., pre-to post-treatment) changes in inflammation and clinical outcomes. It is possible that these null results are due to insufficient power to detect such an effect. Relatedly, as post-treatment inflammation was not significantly decreased relative to baseline, the null result involving long-term changes in inflammation may be due to insufficient variation to detect such an association. Alternatively, it is possible that neither acute nor long-term changes in inflammation were significant individual predictors of clinical response because the relationship is conditional on another unknown variable or variables, or because it is the interaction between acute and long-term processes that affect clinical symptomatology (i.e., acute inflammatory changes differentially impact treatment outcomes based on the overall effect of ECT on inflammation at post-treatment).

The present study found evidence to support the hypothesis that the interaction between the initial inflammatory response to ECT and its long-term inflammatory outcomes predict post-treatment clinical outcomes, however, the direction of this effect was not entirely as predicted. Our results indicated that larger acute increases in IL-6 were associated with better improvement in affective and cognitive symptoms of depression, in individuals with relatively *higher* concentrations of IL-6 at post-treatment, whereas no significant association was detected in individuals with lower post-treatment IL-6. Larger initial increases in CRP were similarly associated with more improvement in neurovegetative symptoms of depression in those with relatively higher CRP at post-treatment but not in those with lower post-treatment CRP. Notably, these results were significant over and above any effects of pre-treatment inflammation, age, sex or BMI. We had originally theorized that the acute inflammatory reaction to ECT would indicate a healthy initial stress response that might subsequently initiate protective and reparative processes ultimately contributing to clinical efficacy. Though we had theorized that initiation of such protective and reparative processes would be reflected by relatively *decreased* post-treatment levels of inflammation, this likely is not the case. Because higher initial spikes in inflammation would necessitate correspondingly larger decreases for post-treatment levels to return to baseline, such subjects may be more likely to be among those with relatively higher IL-6 and CRP levels at post-treatment. Notably, other individuals who did not have as high of an initial spike may have also had higher post-treatment IL-6 levels for other reasons that would relate to poorer treatment outcomes (e.g., chronic inflammation and low stress reactivity), thus providing the contingencies necessary for an association to be detected at that level of the moderator. Another possibility is that, rather than indicating dysfunction in negative feedback processes, slightly elevated inflammation at post-treatment (in conjunction with a larger initial inflammatory response) could be indicative of a healthy (as opposed to blunted) stress response. Though the magnitude of the inflammatory response to ECT should become decreased after repeated exposures, an inflammatory response would still occur for subsequent administrations (Fluitman et al., 2011; Järventausta et al., 2017). As such, those with relatively higher levels of inflammation at post-treatment may show slightly more residual inflammation from the most recent ECT index, while those with relatively lower post-treatment inflammation may show a more blunted inflammatory response either due to dysfunction at baseline or habituation to ECT. In effect, relatively higher post-treatment inflammation may potentially reflect more engagement of stress-reactive repair processes.

The following limitations should be considered when interpreting these results. First, these findings should be considered preliminary due to the limited sample size – particularly for analyses including depression symptom subscales due to missing individual item-level ratings for several TRD subjects, and for cross-sectional comparisons between HC and TRD. Noting that this study was not explicitly designed to detect differences between diagnostic groups, but rather to the extent of ECT-related normalization in patients. Second, though a HC group was included, there was no TRD control group (due to ethical concerns, given the clinical severity of these subjects), which limits our ability to draw causal conclusions regarding the influence of ECT. Further, for clinical reasons, there were differences in ECT parameters amongst TRD subjects, including total duration of treatment and laterality of electrode placement. However, these factors were not related to measures of inflammation or clinical outcomes in the present study, with the exception of lead placement laterality, which was related to global response status but not other clinical outcomes. In addition, as post-treatment blood was collected only once within one week of ECT completion, our ability to understand changes in the inflammatory response to an ECT index administration over time is limited.

Despite these limitations, this study provides preliminary evidence that patterns of change in inflammation associated with ECT may relate to its clinical outcomes. Future studies further probing the ways in which both early/acute and long-term inflammatory effects from repeated ECT may interact and influence clinical treatment outcomes are warranted. In particular, future research should include examination of changes in specific types of depressive symptoms, rather restricting outcomes to global symptom change. In addition to the symptom clusters explored in the present study, others should consider examining changes in other relevant symptom domains and constructs – particularly those shown to be related to both depression and inflammation such as anhedonia, reward processing, anxiety, and threat sensitivity (Lucido et al., 2021; Milaneschi et al., 2021; O'donovan et al., 2013; Renna et al., 2018). Improved understanding of how ECT affects changes in peripheral inflammation and how such changes relate to subsequent clinical outcomes could provide important insights into mechanisms of treatment response and resistance in major depression, both in relation to ECT and more broadly. Such research has the potential to inform the development of more personalized treatment approaches to optimize outcomes in this otherwise extremely difficult-to-treat population.

## CRediT authorship contribution statement

**Christina M. Hough:** Conceptualization, Formal analysis, Visualization, Writing – original draft, Writing – review & editing. **Jennifer L. Kruse:** Conceptualization, Funding acquisition, Methodology, Writing – review & editing. **Randall T. Espinoza:** Conceptualization, Funding acquisition, Investigation, Methodology, Supervision, Writing – review & editing. **John O. Brooks:** Investigation, Writing – review & editing. **Eliza J. Congdon:** Data curation, Investigation, Project administration, Writing – review & editing. **Viviane Norris:** Data curation, Investigation, Project administration, Writing – review & editing. **Michelle G. Craske:** Supervision, Writing – review & editing. **Katherine L. Narr:** Conceptualization, Funding acquisition, Investigation, Methodology, Supervision, Writing – review & editing.

## Financial Support

This work was supported by funding provided by the 10.13039/100000025National Institute of Mental Health to Drs. Narr and Espinoza (R01MH092301), Dr. Narr (K24MH102743) and Dr. Kruse (K23MH116127); the National Science Foundation Graduate Research Fellowship Program to Dr. Hough (DGE-1650604); and the Muriel Harris Chair of Geriatric Psychiatry to Dr. Espinoza. No granting or funding agency had a role in the study design and conduct; data collection, management, analysis and interpretation; or manuscript preparation, review, or approval. All authors had full access to all study data and take responsibility for the integrity and accuracy of the data and its analysis. The contents of this publication are solely the responsibility of the authors and do not necessarily represent the official views of the NIH.

## Declaration of competing interest

The authors declare the following financial interests/personal relationships which may be considered as potential competing interests: C.M. Hough reports financial support was provided by 10.13039/100000001National Science Foundation. J.L. Kruse, R.T. Espinoza, K.L. Narr reports financial support was provided by 10.13039/100000025National Institute of Mental Health. R.T. Espinoza reports a relationship with The Journal of ECT that includes: board membership. If there are other authors, they declare that they have no known competing financial interests or personal relationships that could have appeared to influence the work reported in this paper.

## Data Availability

Data will be made available on request.
