# Supplementary material for: Trajectory of peripheral inflammation during index ECT in association with clinical outcomes in treatment-resistant depression
Source: Brain Behav Immun Health. 2024 Dec 15;43:100925. doi: 10.1016/j.bbih.2024.100925 (PMC11743860; doi:10.1016/j.bbih.2024.100925)
Supplement: Multimedia component 1 [file mmc1.docx]

**Supplemental Table 1. Correlations Between Baseline Depression Severity Ratings**

|  | Affective Subscale | Cognitive Subscale | Neurovegetative Subscale |
| --- | --- | --- | --- |
| MADRS Total | *rho*=.668, *p<*.001 | *rho*=.728, *p<*.001 | *rho*=.426, *p=*.024 |
| HDRS Total | *rho*=.693, *p<*.001 | *rho*=.572, *p<*.001 | *rho*=.674, *p<*.001 |

Abbreviations: MADRS = Montgomery-Åsberg Depression Rating Scale; HDRS = Hamilton Depression Rating Scale


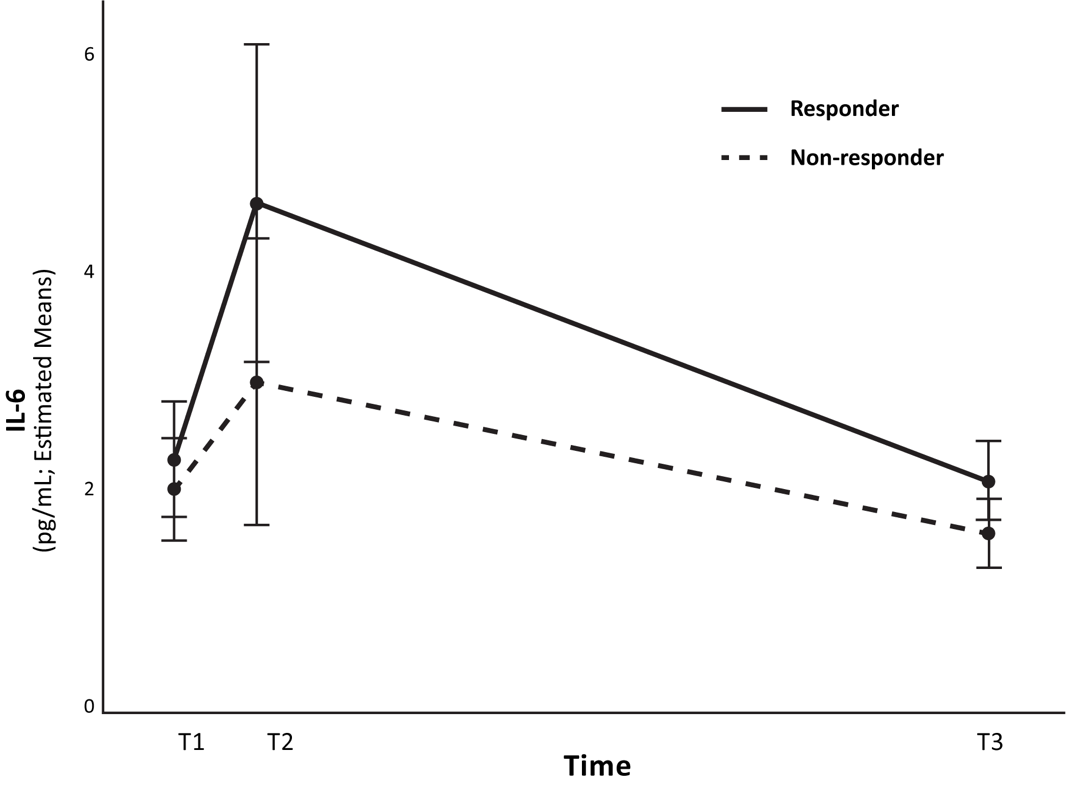


**Supplemental Figure 1. Estimated IL-6 concentrations over time in TRD subjects classified as Responders and Non-responders to ECT.** Error bars represent ± 1 Standard Error; estimated means calculated on average levels of covariates included in the models (age, sex and BMI).
